# Supplementary material for: Tailoring of Ultrasmall NiMnO3 Nanoparticles: Optimizing Synthesis Conditions and Solvent Effects
Source: Molecules. 2024 Oct 13;29(20):4846. doi: 10.3390/molecules29204846 (PMC11510137; doi:10.3390/molecules29204846)
Supplement: Supplementary file 1 [file molecules-29-04846-s001.zip › molecules-3044018-supplementary.pdf]

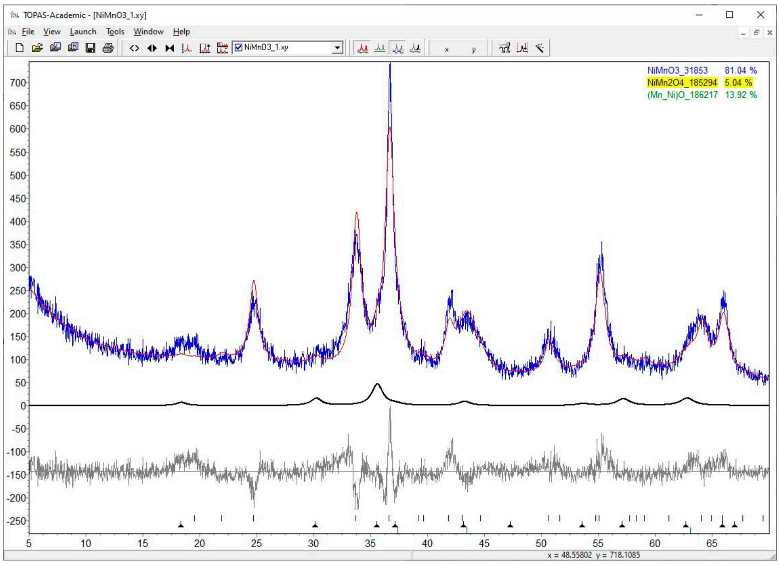

Figure S1. Phase composition of the as-prepared sample $NiMnO_3$. The red line indicates the calculated model. The difference between both values and the line diagram of phase are presented in the lower portion of the graph and indicated by the black line.

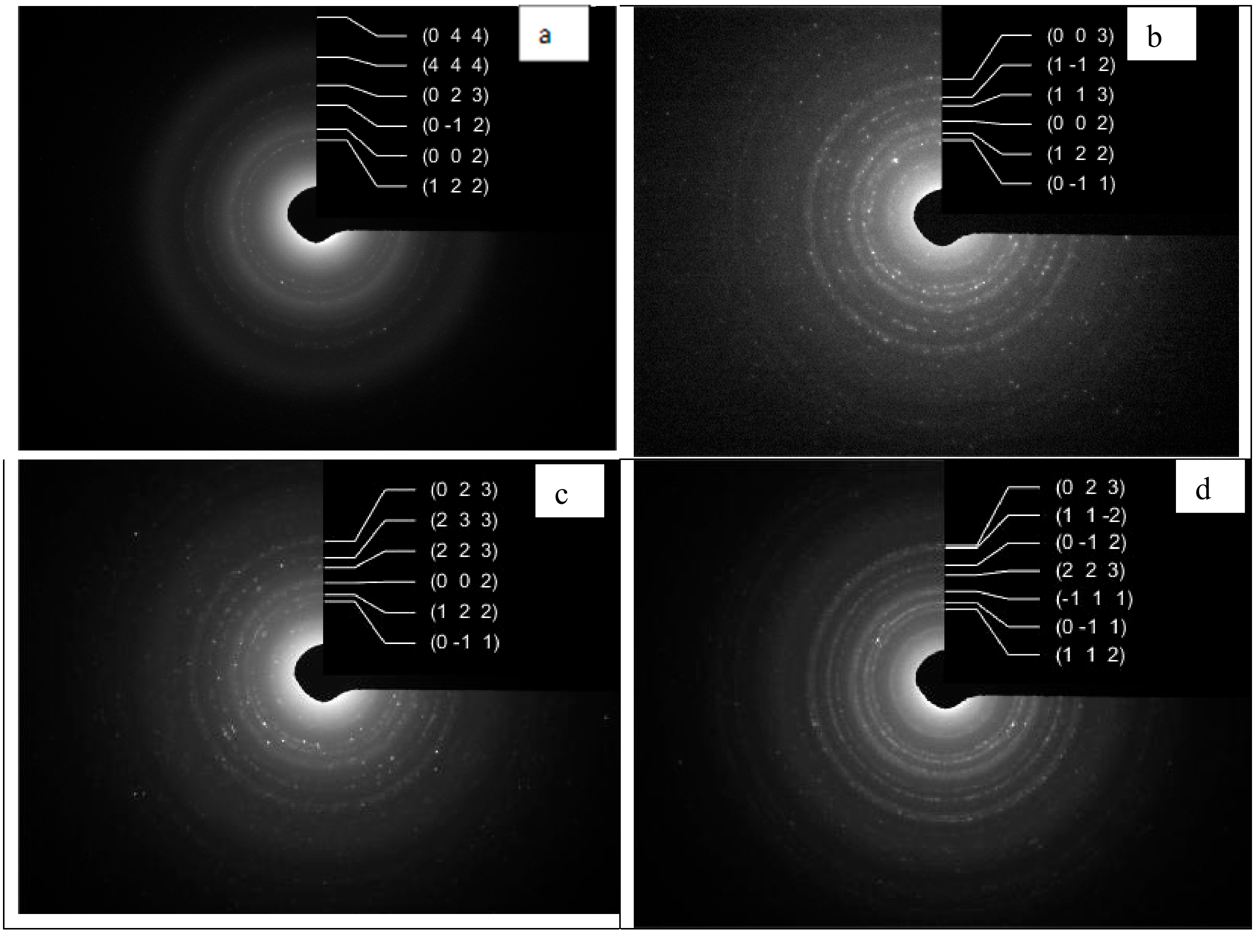

Figure S2. SAED patterns of $NiMnO_3$ after sonication ($NiMnO_3$-NMP (a), $NiMnO_3$-DMSO (b), $NiMnO_3$-DMF (c) and $NiMnO_3$-H_2O (d)) resolve multiple crystallographic planes that correspond to $NiMnO_3$

Table S1. Physical characteristics of the studied liquids

Properties	H_2O	DMFA	DMSO	NMP
T, boiling °C	100	178	189	202
Cp, J/(g·K)	4.18	2.06	1.86	3.11
η, Pa·s	1.020	0.795	2.475	1.663
σ, N/m²	72.0	36.2	43.0	40.7
M, g/mol	18	73	78	99
ρ, g/cm³	1.00	0.94	1.10	1.03
Gas solubility in liquids, мM:				
O_2	1.22	4.78	2.5	3.26
N_2	0.71	2.85	1.17	2.23

Table S2. A comparison of results obtained here with that of previous works

Synthesis method	Conditions	Composition	Crystallinity	Cell parameters of $NiMnO_3$, Å	Particle size. nm	Reference
Hydrothermal from the oxides	600 °C; 1000 atm.	$NiMnO_3$ NiO MnO_2	Good	a=4.905 c=13.59	-	[5]
Hydrothermal with ammonia	pH=8 140 °C for 4 h; 400 °C for 4 h	$NiMnO_3$	Average	-	41	[6]
Hydrothermal with urea	160 °C for 4-8 h; 500 °C for 4 h	$NiMnO_3$ NiO Mn_5O_8	Good	-	-	[7]
	180 °C for 12 h	$NiMnO_3$	Very poor	-	10-200	[8]
Coprecipitation by $NaHCO_3$	pH=8,5 70 °C for 12 h; 430 °C for 5 h	$NiMnO_3$ impurities	Average	-	-	[9]
Coprecipitation by NaOH	pH=10 450 °C for 8 h	$NiMnO_3$	Good	a=4.921± 0.002 c=13.658±0.001	10; 40	This paper

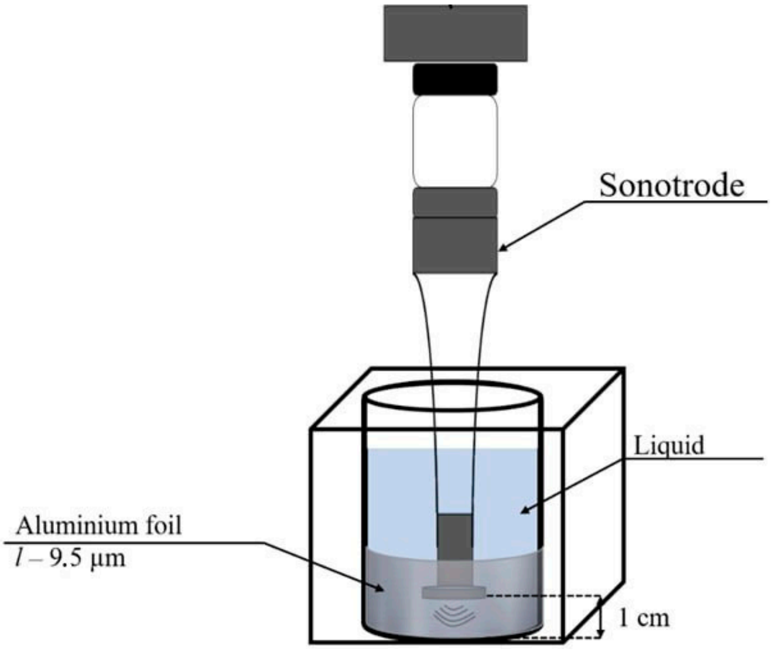

Figure S3. Schematic setup for studying the influence of the solvent on the ultrasonic treatment process of aluminum

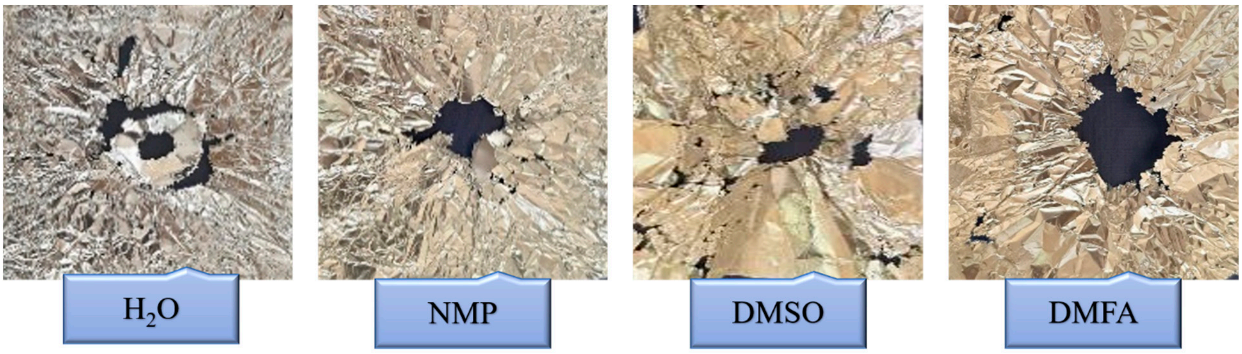

Figure S4. Aluminium foil after ultrasonic treatment
